# Long-range transcriptional regulation by the p110 CUX1 homeodomain protein on the ENCODE array

**DOI:** 10.1186/1471-2164-14-258

**Published:** 2013-04-16

**Authors:** Charles Vadnais, Arif A Awan, Ryoko Harada, Pier-Luc Clermont, Lam Leduy, Ginette Bérubé, Alain Nepveu

**Affiliations:** 1 Goodman Cancer Centre, McGill University, 1160 Pine avenue West, Montreal, Quebec H3A 1A3, Canada; 2 Department of Biochemistry, McGill University, 1160 Pine avenue West, Montreal, Quebec H3A 1A3, Canada; 3 Department of Medicine, McGill University, 1160 Pine avenue West, Montreal, Quebec H3A 1A3, Canada; 4 Department of Oncology, McGill University, 1160 Pine avenue West, Montreal, Quebec H3A 1A3, Canada

**Keywords:** ChAP-chip, Chromatin immunoprecipitation, ENCODE and promoter microarrays, Expression profiling, shRNA, Lentivirus overexpression, Transcriptional activation and repression, Regulation at a distance, Cut homeobox 1 (CUX1)

## Abstract

**Background:**

Overexpression of the Cut homeobox 1 gene, *CUX1*, inversely correlates with patient survival in breast cancers. Cell-based assays and molecular studies have revealed that transcriptional regulation by *CUX1* involves mostly the proteolytically processed p110 isoform. As there is no antibody specific to p110 CUX1 only, an alternate strategy must be employed to identify its targets.

**Results:**

We expressed physiological levels of a tagged-p110 CUX1 protein and performed chromatin affinity purification followed by hybridization on ENCODE and promoter arrays. Targets were validated by chromatin immunoprecipitation and transcriptional regulation by CUX1 was analyzed in expression profiling and RT-qPCR assays following CUX1 knockdown or p110 CUX1 overexpression. Approximately 47% and 14% of CUX1 binding sites were respectively mapped less than 4 Kbp, or more than 40 Kbp, away from a transcription start site. More genes exhibited changes in expression following CUX1 knockdown than p110 CUX1 overexpression. CUX1 directly activated or repressed 7.4% and 8.4% of putative targets identified on the ENCODE and promoter arrays respectively. This proportion increased to 11.2% for targets with 2 binding sites or more. Transcriptional repression was observed in a slightly higher proportion of target genes. The CUX1 consensus binding motif, ATCRAT, was found at 47.2% of the CUX1 binding sites, yet only 8.3% of the CUX1 consensus motifs present on the array were bound *in vivo*. The presence of a consensus binding motif did not have an impact on whether a target gene was repressed or activated. Interestingly, the distance between a binding site and a transcription start site did not significantly reduced the ability of CUX1 to regulate a target gene. Moreover, CUX1 not only was able to regulate the next adjacent gene, but also regulated the gene located beyond this one as well as the gene located further away in the opposite direction.

**Conclusion:**

Our results demonstrate that p110 CUX1 can activate or repress transcription when bound at a distance and can regulate more than one gene on certain genomic loci.

## Background

The last decade has seen significant advances in the field of transcription. The discovery of nuclear histone acetyltransferases (HATs) in the mid nineties has literally opened a new field of investigation into post-translational modifications that target histones and modulate the chromatin state either locally or over large genomic loci. The multiple types of modifications that take place on specific histone residues and the regulatory cascades that can be triggered in this manner led investigators to propose that a "histone code" regulates gene expression in a manner reminiscent of the genetic code translating nucleic acid coding sequences into protein sequences [1,2]. In parallel, a number of novel experimental approaches have contributed to move the transcription field from a gene-by-gene approach focused on core promoters to a genome-wide non-biased approach that enables us to study large numbers of transcriptional targets as well as the mechanisms by which these targets are regulated [3]. Recent tools in our arsenal include the increasing availability of genomic microarrays [4], siRNA-mediated gene knockdown [5], more efficient virus-based gene delivery systems [6,7], and high-throughput sequencing [8]. Importantly, the "rediscovery" of chromatin immunoprecipitation combined with the development of microchip arrays containing large numbers of genomic sequences has opened new horizons. Indeed, chromatin immunoprecipitation was first described in the mid eighties by the group of John T. Lis who used this assay to show that RNA polymerase II molecules were already present at the 5' end of the hsp70 gene in uninduced cells and that heat shock somehow enabled transcription elongation to take place [9]. Curiously, the method was not applied to specific transcription factors before another decade [10]. Interestingly, the genomic microarray that was designed as part of the ENCODE project provided a sampling of the human genome that can be interrogated to define the distribution types of transcriptional regulation of specific transcription factors [11]. The information thus gathered has forced us to reconsider our original understanding of basic mechanisms of transcriptional regulation [12]. For example, a common belief was that a specific transcription factor could bind to a few dozen genes whose core promoters contain its consensus binding site as defined *in vitro*, and once recruited to a promoter could almost single-handedly regulate transcription [13]. We now know that c-MYC binds to approximately 20% of gene promoters and is also capable of regulating genes at a distance [14-16]. Another major conceptual advance concerns the criteria to define a transcriptional target. Experimental evidence typically included the presence of a consensus binding motif within a core promoter, *in vitro* binding assays and luciferase reporter assays. While these assays are still employed, it is clear that they cannot provide definitive evidence that a transcription factor regulates a specific gene. Additional evidence must also include chromatin immunoprecipitation assays to demonstrate "in vivo" DNA binding, and change in expression of the endogenous gene target in response to the knockdown and/or overexpression of the transcription factor.

Cut homeobox 1 (*CUX1*) has previously been called CCAAT-displacement protein (CDP), CDP/Cut and Cut-like 1 (*CUTL1*). *CUX1* encodes two main isoforms that exhibit different DNA binding and transcriptional properties (reviewed in [17]). The full-length protein, p200 CUX1, is a very abundant protein that binds DNA with extremely fast kinetics [18]. In mid-G1 phase, 1% to 10% of p200 CUX1 is proteolytically processed by a nuclear isoform of cathepsin L to produce the p110 CUX1 isoform [19,20]. This shorter isoform can stably interact with DNA and, depending on promoter-context, can function as transcriptional repressor or activator [21,22]. The expression and activity of p110 CUX1 are tightly regulated in a cell cycle-dependent manner, mostly through phosphorylation-dephosphorylation by cyclin A/Cdk2, cyclin A/Cdk1 cyclin B/Cdk1, and Cdc25A, as well as proteolytic processing by nuclear cathepsin L and a caspase-like protease [19,20,23-27]. These post-translational modifications circumscribe the transcriptional activity of p110 CUX1 to the period between mid-G1 to sometimes in G2. In contrast to p110 CUX1, the DNA binding activity of p200 CUX1 is constant throughout the cell cycle [19]. Its transcriptional activity, if any, would be limited to the "CAATT-displacement activity", a mechanism of passive repression involving competition for binding site occupancy [18].

Homozygous inactivation of *Cux1* in mice causes perinatal lethality in a large proportion of animals due to delayed lung development and associated respiratory failure [28]. Surviving mice are usually male and exhibit growth retardation, disrupted hair follicle morphogenesis, purulent rhinitis, infertility, cachexia, and reduction of B and T cell content in bone marrow and thymus, respectively [28-30]. In transgenic mouse models, overexpression of CUX1 generated various cancer-associated disorders depending on the specific isoform and tissue type expression. These include multi-organ organomegaly, glomerulosclerosis and polycystic kidneys, pre-cancerous lesions in the liver, myeloproliferative-disease-like myeloid leukemias and mammary tumors sometimes associated with lung metastasis [31-36]. Cell-based assays demonstrated a role for CUX1 in cell cycle progression and cell proliferation [27,37], strengthening of the spindle assembly checkpoint [38], cell migration and invasion [22,39-41], resistance to apoptotic signals [42], and dendrite branching and spine development in cortical neurons [43]. Which CUX1 isoform(s) is active in these processes cannot be determined from siRNA or shRNA-mediated knockdown approaches, however, in overexpression studies the p110 CUX1 isoform was shown to regulate transcription of genes involved in cell cycle progression, DNA damage response, spindle assembly checkpoint and cell motility.

Many specific transcription factors are able bind to genomic sites that are far away from TSS. These studies also revealed that only about up to 10% of putative transcriptional targets showed evidence of regulation in response to changes in transcription factor concentrations [44-46]. Whether CUX1 binds preferentially to core promoter sequences, like E2F1, or whether it can also bind at a distance from TSS, like c-Myc, has not been determined [14,15]. Also, what proportion of all CUX1 targets is regulated in response to overexpression or silencing of CUX1 is not known. To begin to address these questions, we have performed ChAP-chip using ENCODE and promoter microarrays. Putative targets were validated in independent ChIP followed by q-PCR, while regulatory effects were measured in expression profiling experiments and confirmed by RT-qPCR. The results show that CUX1 binds to a large number of genomic sites that are located far away from a TSS and can regulate genes at a distance even when another gene is located in the intervening region.

## Results

### Strategy to identify p110 CUX1 binding sites

**Figure 1:**
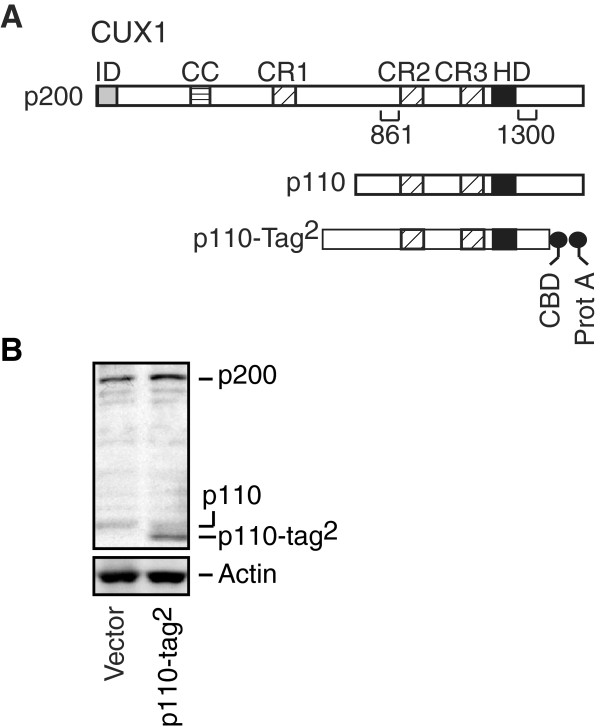
**Expression of CUX1 Recombinant Proteins.** (**A**) Schematic representation of CUX1 proteins with some of the functional domains: ID, inhibitory domain; CC, coiled-coil; CR1, CR2 and CR3, Cut repeat 1, 2 and 3; HD homeodomain; CBD, calmodulin binding domain; Prot A, protein A. The regions recognized by the 861 and 1300 antibodies are shown. (**B**) Hs578t cells were infected with a retroviral vector to establish a population of cells stably expressing a recombinant p110 CUX1 protein with two tags at its C-terminus, p110 CUX1-Tag^2^. A population stably carrying the empty vector was used as a control. Nuclear extracts were prepared from each population of cells and analyzed by Western blot using the 861 and 1300 CUX1 antibodies.

The overall goal of the present study was to define the modes of transcriptional regulation by CUX1 and, in particular, determine whether CUX1 can regulate genes at a distance. As detailed in the introduction, previous transcriptional studies and cell-based assays have implicated the p110 CUX1 isoform in transcriptional activation and repression of target genes. Since p110 CUX1 is generated by proteolytic processing, its primary sequence is included in the full-length CUX1 protein sequence. Consequently, all available antibodies that bind to p110 CUX1 also recognize p200 CUX1. Our strategy to identify in vivo binding sites for p110 CUX1 was to isolate chromatin by two different methods. First, we purified chromatin by tandem affinity purification (TAP) using a population of Hs578t cells stably expressing moderate levels of a p110 CUX1 protein with two epitope tags at its C-terminus, p110-Tag^2^ (Figure 1A and B). Chromatin isolated in this manner as well as total chromatin (input) were used in hybridizations on the NimbleGen HG17 ENCODE high density oligonucleotide tiling array. Secondly, binding sites identified in the microarray were then validated by performing independent ChIP in the parental Hs578t cells using CUX1 antibodies, 861 and 1300 (Figure 1A). Importantly, these cells express endogenous CUX1 proteins only. The strategy of chromatin affinity purification (ChAP) followed by microarray analysis (ChAP-chip) has previously been validated [47], and described in detail [48].

### Distribution of CUX1 binding sites on the ENCODE array

Using a stringent false discovery rate (FDR = 0.05), 513 CUX1 binding sites were identified on the ENCODE array (Table 1). The recruitment of CUX1 to 23 out of 25 genomic sites (92%) was validated in quantitative-PCR assays using chromatin that was independently obtained from Hs578t cells by immunoprecipitation with CUX1 antibodies (Table 1). 79.6% of probes on the ENCODE array derive from transcribed genomic regions. 70.9% of CUX1 binding sites were located within transcribed regions, indicating a 1.6-fold enrichment in non-transcribed regions. In comparison, data obtained from ChIP on the ENCODE platform [14] for c-MYC reveals a 1.56 fold enrichment in non-transcribed regions while E2F1 showed a strong enrichment for transcribed regions (Table 2).

**Table 1:** CUX1 binding sites on the ENCODE array

# of binding sites	513
Average site width (bp)	503
Sites tested in qPCR	25
Validation rate	92%
Validation rate (with consensus)	100%
Validation rate (no consensus)	90%

Number and average width of CUX1 binding sites identified on the NimbleGen HG17 ENCODE array using chromatin purified from Hs578t cells. Also shown are the number and percentage of binding sites that were validated in an independent ChIP experiment. The validation rate is also shown independently for sites that contained the ATCRAT consensus sequence as well as for sites that did not.

**Table 2:** Distribution of CUX1, Myc and E2F1 binding sites in transcribed and non-transcribed regions

	**Encode platform**	**CUX1**	**c-MYC**	**E2F1**
Number of binding sites		513	172	204
Non-transcribed regions	20.4%	28.1%	28.5%	5.9%
Transcribed regions	79.6%	70.9%	71.5%	94.1%
Enrichment in un-transcribed regions		1.61	1.56	0.24
P Value		0.0018	0.1333	<0.0001

Number of CUX1, C-Myc and E2F1binding sites in transcribed and un-transcribed regions. Also indicated are the fold enrichment in transcribed regions. P Values are calculated using a Fisher's exact test.

**Figure 2:**
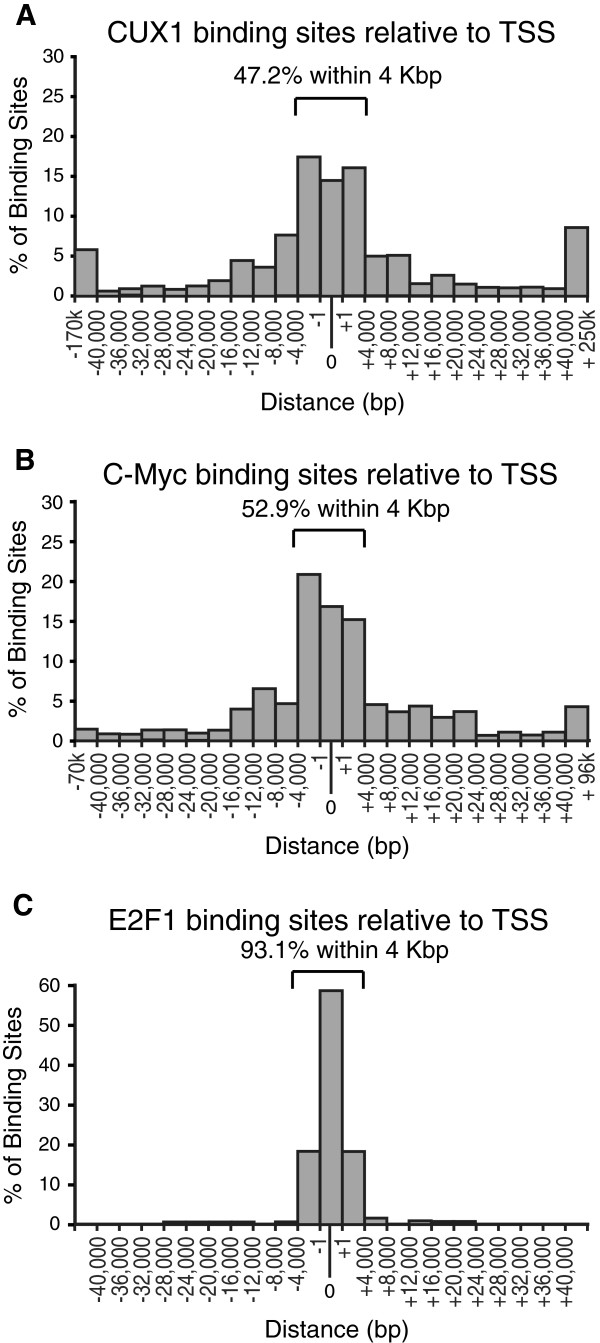
**Distribution of CUX1, C-Myc and E2F1 Binding Sites Relative to Transcription Start Sites.** (**A**) Percentage of CUX1 binding sites located at various distances from the closest transcription start site. The "0" column indicates genes where the CUX1 binding site overlaps the start site. (**B**) Location of C-Myc binding sites as per **A**. (**C**) Location of E2F1 binding sites as per **A**.

Mapping of CUX1 binding sites relative to transcription start sites (TSS) generated a bell-shaped curve of low height around TSS (Figure 2A). 14.2% of all binding sites overlapped a TSS, and an additional 17% and 16% of binding sites were respectively located in the 4 Kbp region upstream and downstream of a TSS. The number of binding sites gradually declined with increasing distance. Yet, over 6% and 8% of binding sites were situated at more than 40,000 bp upstream or downstream, respectively, from the closest TSS. 53% of CUX1 binding sites are located more than 4,000 bp away from a TSS and approximately 14% of all CUX1 binding sites are situated at more than 40,000 bp from a TSS.

We compared the distribution of CUX1 binding sites with those of 3 randomly generated sets of binding sites, as well as those of c-Myc and E2F1 using the data of Bieda et al., 2006 [14] (Figure 2B and C). We note that the distributions of randomly generated sets of binding sites exhibited flatter bell-shaped curves around TSS (Additional file 1: Figure S1). We conclude that the higher frequency of CUX1 binding sites close to TSS reflects the preferential recruitment of CUX1 to promoter regions. The same cannot be said regarding the binding sites that are located at more than 40 Kbp from TSS, since the same proportions of randomly generated binding sites were located in these regions.

In contrast to CUX1 and c-Myc, the E2F1 transcription factor was found to bind almost exclusively to the region immediately adjacent to TSS. The preference of E2F1 to core promoter regions led the authors to posit that E2F1 is recruited via protein interactions with components of the general transcription machinery [14]. The wider distribution of binding sites observed for CUX1 and c-Myc is also observed for other transcription factors [15,49,50] (Additional file 2: Figure S2A-C), while other factors show a preference for TSS similarly to E2F1 (Additional file 2: Figure S2D-E). Yet other factors show different patterns of binding, such as Pax8, which exhibits preference for non-promoter CpG islands and a tendency to bind in the 10–100 Kbp range rather than close to the TSS of genes [51].

### Binding of CUX1 to distant regulatory elements

We compared the location of CUX1 binding sites that are more than 4 Kb from the nearest TSS to DNAse hypersensitivity mappings and ChromHMM data in human mammary epithelial cells from published datasets. DNAse hypersensitivity sites have been used as markers of regulatory DNA elements such as enhancers, silencers, insulators and locus control regions [52-55]. ChromHMM is a computational method that compiles data from histone modification mappings and integrates them to predict genomic elements such as enhancers [56]. This analysis revealed that respectively 19.2% and 22.1% of distantly located CUX1 binding sites are present within 1 kb of a DNAse hypersensitivity site and of an enhancer predicted (Table 3). Both of these proportions are greater than what is seen for randomly distributed binding sites. However, there was no enrichment of CUX1 binding sites in proximity of insulator elements (Table 3). These results are in agreement with the notion that CUX1 can perform some regulatory functions when binding at a distance from transcription start sites.

**Table 3:** A fraction of CUX1 binding sites locate close to enhancer elements and DHS sites

**Type**	**CUX1**	**Random**	**Fold difference**	**P Value**
DHS	19.2%	12.9%	1.49	0.0109
Enhancers	22.1%	15.2%	1.45	0.0100
Insulators	4.43%	4.40%	1.01	1.0000

Percentages of CUX1 binding sites located more than 4 Kbps away from a TSS but within 1 Kbp of the indicated type of genomic element. Percentages are shown for a set of randomly generated binding sites of the same size distribution as CUX1. P Value is calculated using a Fisher's exact test. See Methods for information on the datasets used.

### Detection of CUX1 binding sites and consensus binding motif on promoter arrays

Promoter microarrays are useful because they enable one to interrogate easily over 30,000 gene promoters. A limitation is that only a limited amount of promoter sequences can be included for each gene, precluding the detection of far away binding sites that could play a role in transcriptional regulation. Based on the localization of CUX1 binding sites on the ENCODE array, we calculated that between 17.2% to 26.6% of CUX1 binding sites would be identified on commercially available promoter arrays (Table 4). However, since for many distant CUX1 binding sites another binding site is also present close to the transcription start site, we estimated that between 44.6% to 58.5% of gene targets would be identified on distinct promoter arrays (Table 5). In contrast, as E2F1 is targeted to transcription start sites, between 80.4% to 85.8% of E2F1 binding sites would be expected to be identified on a promoter array.

**Table 4:** Binding sites and target genes predicted to be identified in promoter arrays

**Platform**	**Promoter array boundaries**	**% of Binding sites predicted in promoter array**		
		**CUX1**	**C-Myc**	**E2F1**
Nimblegen	−3.5 kb to + 0.75 kb	17.2%	26.8%	80.4%
Agilent	−5.5 kb to + 2.5 kb	23.4%	34.3%	84.3%
Affymetrix	−7.5 kb to + 2.45 kb	26.6%	34.9%	85.8%

Percentages of CUX1, C-Myc and E2F1 binding sites that were identified on the ENCODE array and that are located within the boundaries of promoters on various promoter array platforms.

**Table 5:** Binding sites and target genes predicted to be identified in promoter arrays

**Platform**	**Promoter array boundaries**	**% of Target genes predicted in promoter array**		
		**CUX1**	**C-Myc**	**E2F1**
Nimblegen	−3.5 kb to + 0.75 kb	44.6%	36.0%	90.2%
Agilent	−5.5 kb to + 2.5 kb	57.1%	45.9%	92.2%
Affymetrix	−7.5 kb to + 2.45 kb	58.5%	48.3%	92.2%

Percentages of CUX1, C-Myc and E2F1 target genes that are identified on the ENCODE array and whose binding site are located within the boundaries of promoters on various promoter array platforms.

We verified these predictions by performing a ChAP-chip experiment using the Nimblegen promoter microarray. Total chromatin (input) as well as purified chromatin from Hs578t cells expressing p110 CUX1-Tag2 were used in hybridization on the promoter array of NimbleGen. Using a stringent false discovery rate (FDR = 0.05), 5828 CUX1 binding sites were identified on 4706 gene promoters (Table 6). The recruitment of CUX1 to 25 out of 25 genomic sites (100%) was validated in quantitative-PCR assays using chromatin that was independently obtained from Hs578t cells by immunoprecipitation with CUX1 antibodies (Table 6). The vast majority of target genes (83.7%) contained only one CUX1 binding site, yet a sizable fraction contained 2 or more binding sites (Table 6).

**Table 6:** CUX1 binding sites on the promoter array

**Genes on array**	**20593**	**Number of sites/gene**	**Number of genes**
CUX1 Binding sites	5828	1	3942
Genes bound by CUX1	4706	2	643
Average Site Width (bp)	503	3	90
Sites tested in qPCR	25	4	23
Validation rate	100%	5+	8

Columns 1 and 2: Number and average width of CUX1 binding sites identified on the NimbleGen HG18 Human Promoter Array using chromatin purified from Hs578t cells. Also shown are the number and percentage of binding sites that were validated in an independent ChIP experiment.

Columns 3 and 4: Number of genes with the indicated number of CUX1 binding site. Note that validation was performed on 14 and 11 sites that contained or not an ATCRAT motif. All of them were validated.

According to the predictions shown in Table 5, 44.6% of CUX1 target genes should be identified on the promoter array from Nimblegen. We calculated the proportion of ENCODE genes with a CUX1 binding site that were also identified as putative targets of CUX1 in the promoter array. When we considered all 513 CUX1 binding sites and 445 adjacent ENCODE genes, we found that 92 genes (21%) were identified in the promoter array (Table 7, third column). When we considered only the 85 ENCODE genes that were regulated in response to changes in CUX1 levels (see below), we found that 27 genes (32%) were identified as putative target of CUX1 in the promoter array (Table 7, third column).

**Table 7:** Binding sites and target genes predicted to be identified in promoter arrays

	**Total**	**Identified on Nimblegen promoter array**
All Genes on the ENCODE Array	445	92 (21%)
Regulated Genes on the ENCODE Array (1.25)	85	27 (32%)
Regulated Genes on the ENCODE Array (1.5)	26	8 (31%)

The second column shows the number of genes on the ENCODE. The third column shown the number and percentage of these genes that were also identified on the Nimblegen promoter array.

The CUX1 consensus binding site, ATCRAT (where R = C or A), was found to be present at 47.2% of the 5828 bound genomic sites (Table 8). This frequency was judged to be significant as the CUX1 consensus binding site was found to be present in only 17.5% of 5828 randomly chosen regions of equal size. Notably, the GC content between bound and unbound regions is practically identical, and thus cannot account for the difference in binding site occurrence (Table 8). Yet, only 8.3% (3633/43778) of the CUX1 consensus sites present on the array were bound *in vivo*. We conclude that the CUX1 consensus binding site plays a role in the recruitment of CUX1 at specific genomic locations, but the presence of a consensus site is not sufficient.

**Table 8:** CUX1 consensus binding sites and bound genomic regions

	**Regions**	**Regions with consensus**	**% with consensus**	**GC Content**
Bound Regions	5828	2749	47.2%***	47.3%
Unbound Regions	5828	1020	17.5%	47.0%

Columns 2–4, occurrence of the CUX1 consensus binding site, ATCRAT (where R = C or A), within the 5828 genomic regions bound by CUX1 on the promoter array. To calculate the p value, an equal number of randomly chosen regions of equal width was searched for the presence of the CUX1 consensus binding site: ***: p < 0.001. Column 5 shows the GC content of bound and unbound regions.

### Identification of binding motifs in genomic regions bound by CUX1

We envisioned that interactions with other transcription factors play an important role in recruiting CUX1 to specific locations. In agreement with this notion, functional analysis revealed distinct sets of cellular functions among gene targets that contain an ATCRAT consensus and those that do not (Tables 9 and 10). To further test the possibility that CUX1 may interact with other factors, we investigated the presence of binding motifs other than that of CUX1 using the MEME suite of analysis tools (meme.nbcr.net/). We first tested the reliability of the tool by using it to find motifs in the sequences of CUX1 BS in which we had independently determined that they contained the established ATCRAT consensus. As expected, it identified the ATCRAT consensus as the most enriched motif in the set of sequences, by a vast margin (Table 11, entry 1). We then analyzed binding motifs in the two sets of CUX1 binding sites: those that contained the ATCRAT motif and those that did not. While the size of bound regions varied from 149 to 1107 bp, the average size was 532 and 477 bp, respectively. Interestingly, only one common binding motif was found in the two sets, while the rest of the binding motifs were unique to each set (Tables 11 and 12). Extending the search to the 500 bp regions on either side of bound regions did not highlight other differences between the two sets or reveal additional contributing factors (Tables 13 and 14). These findings support the notion that targeting of CUX1 to specific genomic sites is influenced by protein-protein interactions with other DNA binding proteins.

**Table 9:** Functions of CUX1 target genes that contain a consensus CUX1 binding site

**Functional term**	**Fold enrichment**	**P Value**
Macromolecular complex assembly	1.39	3.1E-04
Microtubule cytoskeleton organization	1.88	6.1E-04
Cytoskeleton organization	1.47	6.6E-04
Response to DNA damage stimulus	1.51	7.2E-04
Negative regulation of programmed cell death	1.49	1.2E-03
Anti-apoptosis	1.68	1.2E-03
Cellular response to stress	1.38	1.4E-03
Cellular macromolecule catabolic process	1.32	1.5E-03
Protein localization	1.28	1.7E-03
Translational elongation	1.97	2.3E-03

Ten most over-represented biological functions of CUX1 targets gene from the promoter array which contain a consensus CUX1 binding sequence (ATCRAT). Overrepresentation is determined using the online DAVID tool.

**Table 10:** Functions of CUX1 target genes that do not contain a consensus CUX1 Binding site

**Functional term**	**Fold enrichment**	**P Value**
Ribonucleoprotein complex biogenesis	2.11	3.3E-06
Translation	1.73	1.1E-05
RNA processing	1.55	1.1E-05
Cell cycle	1.41	5.4E-05
Mitotic cell cycle	1.62	6.1E-05
Ribosome biogenesis	2.17	7.4E-05
Nuclear mRNA splicing, via spliceosome	2.02	8.0E-05
Cell cycle process	1.45	1.9E-04
Establishment of protein localization	1.38	1.9E-04
Translational elongation	2.19	3.0E-04

Ten most over-represented biological functions of CUX1 target genes from the promoter array which do not contain a consensus CUX1 binding sequence (ATCRAT). Overrepresentation is determined using the online DAVID tool.

**Table 11:** Identification of DNA motifs in CUX1 binding sites with the ATCRAT consensus

**Motif**	**Reverse complement**	**E-Value**	**Transcription factors**
ATCRAT	ATYGAT	3.5E-735	Cux1, Pbx1
GGGYGGGR	YCCCRCCC	4.8E-35	Klf4, Klf7, Sp1, Sp4, Zfp281, Zfp740, Egr1
AAATAHW	WDTATTT	1.9E-27	-
CTBCCTS	SAGGVAG	6.30E-26	Spi1, Stat3, Fev, Sfpi1
CWCCDCC	GGHGGWG	6.60E-23	-
DRGGAAA	TTTCCYH	6.20E-21	-
BSTGTGTG	CACACASV	1.20E-20	-
RGAGAAR	YTTCTCY	2.60E-14	-
ACRCWG	CWGYGT	3.70E-14	-
RAAACAAA	TTTGTTTY	1.90E-11	Sox11, Sox4, Foxd3, Foxi1

10 Most enriched DNA motifs found in CUX1 binding sites that contain the ATCRAT CUX1 consensus. The DNA sequences considered in this analysis correspond to the entire regions bound by CUX1 as defined in the microarray. The size of bound regions varies from 149 to 1107 bp (95^th^ percentile) and is 532 bp on average. Proteins with DNA binding motifs highly similar to the motifs are listed in the rightmost column. K = G/T, M = A/C, R = A/G, Y = C/T, S = C/G, W = A/T, B = C/G/T, V = A/C/G, H = A/C/T, D = A/G/T.

**Table 12:** Identification of DNA motifs in CUX1 binding sites without the ATCRAT consensus

**Motif**	**Reverse complement**	**E-Value**	**Transcription factors**
DTATTTW	WAAATAH	3.80E-35	-
CYCCRCCC	GGGYGGRG	4.60E-34	Klf4, Klf7, Sp1, Sp4, Zfp281, Zfp740, Egr1
CAYTTCY	RGAARTG	1.50E-26	Gabpa, Stat1
CACASAS	STSTGTG	3.20E-23	Runx1
DGGAAA	TTTCCH	5.00E-22	Stat1, Nfatc2, Rela, Rel, Fev
CCRCCDCC	GGHGGYGG	6.40E-19	-
GSAGAGR	YCTCTSC	3.90E-17	-
CHGCAGC	GCTGCDG	1.30E-16	Myf, Ascl2
CATTTWM	KWAAATG	2.90E-26	-
DTTTCTS	SAGAAAH	1.70E-13	-

10 Most enriched DNA motifs found in CUX1 binding sites that do not contain the ATCRAT CUX1 consensus. The DNA sequences considered in this analysis correspond to the entire regions bound by CUX1 as defined in the microarray. The size of bound regions varies from 149 to 949 bp (95^th^ percentile) and is 477 bp on average. Proteins with DNA binding motifs highly similar to the motifs are listed in the rightmost column.

**Table 13:** Identification of DNA motifs close to CUX1 binding sites with the ATCRAT consensus

**Entry**	**Motif**	**Reverse complement**	**E-value**	**Match in Table**14	**Factors**
1	CNGCCTCC	GGAGGCNG	2.9E-168	Entry 3	-
2	CTGTARTC	GAYTACAG	2.5E-161	Entry 1	-
3	CAGGCTGG	CCAGCCTG	3.7E-145	-	-
4	AAAWAMAA	TTKTWTTT	6.1E-135	Entry 2	Srf, Elf3, Tcfap2e
5	TGCAGTGR	YCACTGCA	4.6E-115	Entry 6	Zbtb3
6	CCAGCTAC	GTAGCTGG	8.9E-109	Entry 4	-
7	GAGACRGR	YCYGTCTC	1.6E-108	-	-
8	BGYGGTGG	CCACCRCV	2.6E-92	-	-
9	CTCCYGMC	GKCRGGAG	1.7E-86	Entry 5	-
10	CAAAGTGC	GCACTTTG	2.1E-71	Entry 10	-

10 Most enriched DNA motifs found within the 500 bp regions on either side of CUX1 binding sites that contain the ATCRAT CUX1 consensus. Column 5 lists Table 14 entries whose motifs are very similar (max. 1 mismatch). Proteins with DNA binding motifs highly similar to the motifs are listed in the rightmost column. K = G/T, M = A/C, R = A/G, Y = C/T, S = C/G, W = A/T, B = C/G/T, V = A/C/G, H = A/C/T, D = A/G/T.

**Table 14:** Identification of DNA motifs close to CUX1 binding sites without the ATCRAT consensus

**Entry**	**Motif**	**Reverse complement**	**E-Value**	**Match in Table**13	**Factors**
1	CTGTARTC	GAYTACAG	3.9E-170	Entry 2	-
2	AAAAWAMA	TKTWTTTT	6.6E-170	Entry 4	Srf, Elf3, Tcfap2e
3	CMGCCTCC	GGAGGCKG	1.7E-160	Entry 1	-
4	AGTAGCTG	CAGCTACT	6.2E-120	Entry 6	-
5	CTCCWSCC	GGSWGGAG	2.3E-116	Entry 9	-
6	GCRGTGR	YCACYGC	2.6E-109	Entry 5	Zbtb3
7	CCMCRCCC	GGGYGKGG	1.4E-104	-	Klf4, Klf7, Sp1, Sp4
8	AAATTAGC	GCTAATTT	2.4E-93	-	Pdx1
9	GTAGAGAY	RTCTCTAC	2.0E-90	-	-
10	AAAGTGCT	AGCACTTT	2.0E-75	Entry 10	-

10 Most enriched DNA motifs found within the 500 bp regions on either side of CUX1 binding sites that do not contain the ATCRAT CUX1 Consensus. Column 5 lists Table 13 entries whose motifs are very similar (max. 1 mismatch). Proteins with DNA binding motifs highly similar to the motifs are listed in the rightmost column.

### Regulatory effects of CUX1 on putative targets

**Figure 3:**
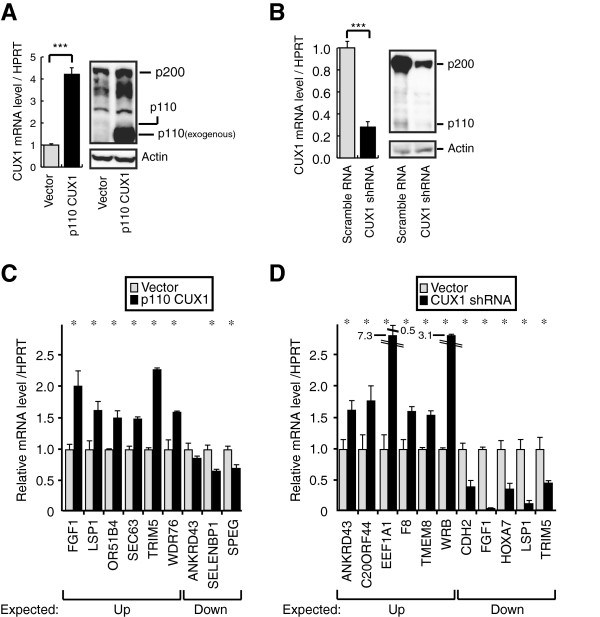
**Overexpression and Knockdown of CUX1 and Expression Profiling Validation.** (**A**) Hs578t cells were infected with a lentiviral vector expressing p110 CUX1 or nothing (vector). RNA and proteins were purified 48 hours post-infection. CUX1 expression was analyzed by RT-qPCR and immunoblotting. (**B**) Hs578t cells were infected with a lentiviral vector expressing CUX1 shRNA or a scrambled RNA. RNA and proteins were purified 5 days after infection. CUX1 expression was analyzed by and RT-qPCR and immunoblotting. (**C**) RNA levels of the indicated genes were measured by RT-qPCR in cells treated as in **A**. Expected up or down indicates regulation that was observed by expression profiling. (**D**) RNA levels of the indicated genes were measured by RT-qPCR in cells treated as in **B**. Expected up or down indicates regulation that was observed by expression profiling. * p<0.05, *** p<0.001 on a Student's T test.

To verify the effect of CUX1 on putative targets, we performed expression profiling on three Hs578t cell populations: cells that had been infected with a retrovirus expressing an shRNA against CUX1, cells infected with a retrovirus expressing p110 CUX1, or cells infected with an empty retrovirus. In each case, replicate microarray hybridizations were carried out such that a p value could be calculated for each difference in gene expression. Results from expression profiling were validated by repeating the infections and performing RT-qPCR analysis on 20 genes whose expression went up or down in response to one treatment or the other (Figure 3). All genes tested in this manner displayed changes in gene expression in the same direction as that observed in the microarray hybridization: genes that were repressed in expression profiling were also repressed when mRNA levels were measured by RT-qPCR. Similar observations were made for genes that were activated. We note, however, that the fold activation or repression calculated by RT-qPCR were not necessarily proportional to the changes observed in microarray hybridization. For example, EEF1A1 and C20ORF44 mRNA were increased respectively 7.3 and 1.8 fold when measured by RT-qPCR, but were increased 1.7 and 1.4 fold in microarray analyses. Some of these differences could be due to the fact that measurements by the two methods were made with RNA prepared from independent experiments. Notwithstanding the differences in magnitude, the effects of CUX1 on gene expression was confirmed for all tested genes.

A total of 445 genes are present on the ENCODE array, and all have a CUX1 binding site located within 213 Kbp of their TSS. Expression profiling results could be matched for 327 of these genes. Using a cut-off of 50% either up or down-regulated and a p value below 0.05, we observed differences in the expression of 26 target genes (7.4%), following changes in CUX1 levels (Table 15). 20 genes responded to CUX1 knockdown, and 6 genes, to p110 CUX1 overexpression (Table 15). Among the 26 regulated target genes, 10 genes (38%) were activated and 16 genes (62%) were repressed by CUX1 (Table 15). Similar proportions of activated and repressed genes were found when a cut-off of 25% change in gene expression was employed (Table 16). These findings confirm that p110 CUX1 can participate in transcriptional activation or repression depending on promoter context.

**Table 15:** Genes on the ENCODE array regulated in response to CUX1 overexpression or CUX1 knockdown (1.5 fold)

**Effect of CUX1 on 327 putative target genes**	**CUX1 Overexpression or Knockdown**	**CUX1 Overexpression**	**CUX1 Knockdown**
Up- or Downregulated	26 (7.4%)	6 (1.7%)	20 (5.7%)
Upregulated	10 (2.9%)	3 (0.9%)	7 (2.0%)
Downregulated	16 (4.6%)	3 (0.9%)	13 (3.7%)

Number and percentage of genes on the ENCODE platform that exhibit a 1.5 fold change in expression following p110 CUX1 overexpression or CUX1 knockdown. "Upregulated by CUX1" are genes whose expression is increased following p110 CUX1 and/or decreased following CUX1 knockdown. Conversely, "Downregulated by CUX1" are genes whose expression is decreased following p110 CUX1 and/or increased following CUX1 knockdown.

**Table 16:** Genes on the ENCODE array regulated in response to CUX1 overexpression or CUX1 knockdown (1.25 fold)

**Effect of CUX1 on 327 putative target genes**	**CUX1 Overexpression or Knockdown**	**CUX1 Overexpression**	**CUX1 Knockdown**
Up- or Downregulated	85 (24.4%)	36 (10.3%)	62 (17.8%)
Upregulated	35 (10.0%)	18 (5.2%)	24 (10.9%)
Downregulated	50 (14.3%)	18 (5.2%)	38 (6.9%)

Number and percentage of target genes on the ENCODE platform that exhibit a 1.25 fold change in expression following p110 CUX1 overexpression or CUX1 knockdown. Genes were analyzed as in Table 15.

Similar results were obtained when we analyzed the expression of putative targets identified on the promoter array. A total of 347 genes, 8.4% of all putative targets for which expression profiling results could be matched, were regulated by CUX1. 287 and 85 genes exhibited regulation in response to CUX1 knockdown or p110 CUX1 overexpression, respectively. 181 (52%) were up-regulated by CUX1 while 167 (48%) were down-regulated by CUX1.

### Effect of distance on transcriptional regulation by CUX1

**Figure 4:**
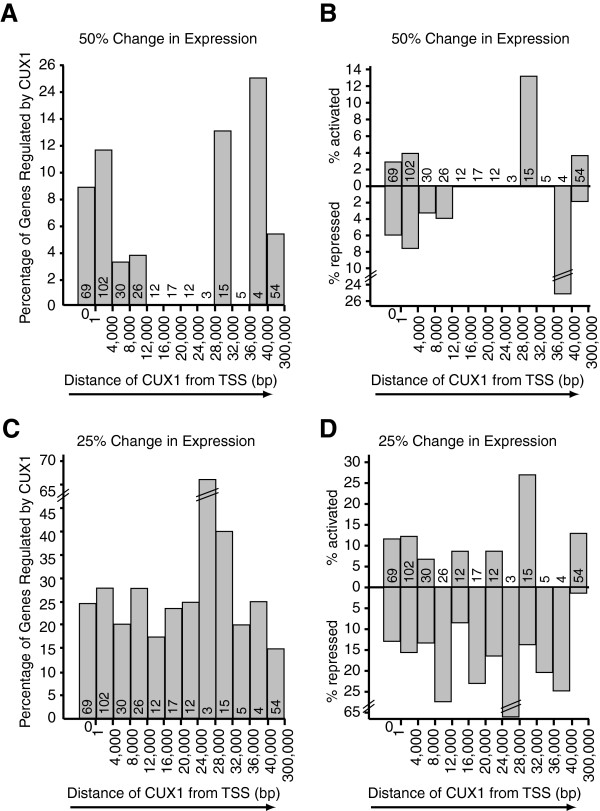
**Effect of Distance on Regulation by CUX1.** (**A**) Genes from the ENCODE array have been organized according to the distance between their transcription start site (TSS) and the closest CUX1 binding site. The "0" column indicates genes where the CUX1 binding site overlaps the start site. The histogram shows, for each interval of distance, the percentage of genes that exhibit a 1.5 fold change in expression following p110 CUX1 overexpression or CUX1 knockdown. The total number of genes within each interval is indicated within each column. (**B**) As in **A**, except that the regulation by CUX1 is expressed as either activation by CUX1 or repression by CUX1. (**C**) As in **A**, but with a threshold of 1.25 fold change in expression. (**D**) As in **B**, but with a threshold of 1.25 fold change in expression.

We noted that CUX1 regulated 7.4% and 8.4% of putative targets from the ENCODE and the promoter arrays, respectively. We next investigated the relationship between the position of a CUX1 binding site relative to a transcription start site and the probability of a gene to be regulated in response to changes in CUX1 levels. When genes were classified according to the distance between the CUX1 binding site and the transcription start site, we did not observe significant difference in the fraction of targets that were regulated by CUX1 (Figure 4A and B). However, we observed much variability in the fraction of regulated genes because the number of genes within some distance intervals were very small. Therefore, to increase the sample size, we repeated the analysis this time using a cut-off of 25% either up or down and a p value below 0.05 (Figure 4C and D). We observed differences in the expression of 62 and 36 genes in response to CUX1 shRNA and CUX1 overexpression, respectively (Table 16). Again, more genes were found to be regulated by CUX1 using the shRNA approach. Among genes that exhibited regulation by CUX1, 35 genes (41%) were activated by CUX1, and 50 genes (59%) were repressed by CUX1 (Table 16). The histogram presenting the percentage of regulated genes versus the distance of CUX1 binding sites to TSS shows that essentially the same proportion of genes are regulated whether CUX1 binds close or far away from the TSS (Figure 4C). Indeed, no statistical difference was observed between genes bound at the TSS and those bound more than 40 Kbp away. We conclude that CUX1 can activate or repress transcription when bound at a distance from a transcription start site.

### Effect of multiple CUX1 sites

The presence of multiple CUX1 binding sites has a modest, yet significant, impact on the probability that a gene is regulated by CUX1. CUX1 regulated 7.9%, 11.2% of genes that contain respectively one or two CUX1 binding sites, respectively (Table 17).

**Table 17:** Number of genes on the promoter array that are regulated in response to CUX1 overexpression or CUX1 knockdown

**Number of sites/target**	**Number of targets**	**Targets with profiling data**	**1.5 fold change**		**1.25 fold change**	
Any #	4706	4140	347	8.4%	1437	34.7%
1	3942	3527	278	7.9%	1182	33.5%
2+	643	613	**69	11.2%	***255	41.6%

Effect of the number of CUX1 binding sites on the probability that target genes exhibit a change in expression following p110 CUX1 overexpression or CUX1 knockdown, depending on the number of CUX1 binding site present in their promoter region. **: P < 0.01, ***:P < 0.001 on a Fisher's exact test vs. genes whose promoter have only 1 CUX1 binding site.

### Effect of gene position on transcriptional regulation by CUX1

**Figure 5:**
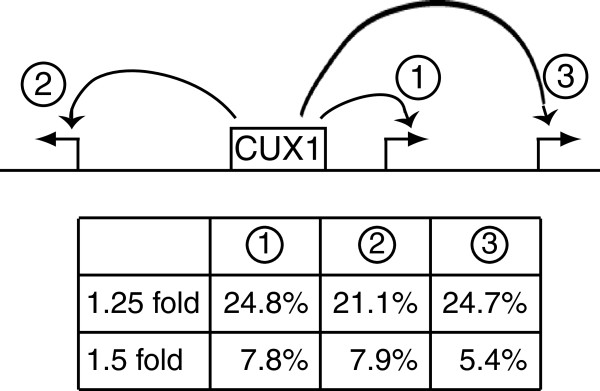
**Relationship Between Gene Position and Regulation by CUX1.** Three types of situations are depicted in the diagram. 1, genes that are the closest to the CUX1 binding site; 2, genes that are further away and in the other direction from the CUX1 binding site; 3, genes that are located further away and are separated by another gene from the CUX1 binding site. For each category, the table shows the percentage of genes that exhibit a 1.25 or 1.5 change in expression following p110 CUX1 overexpression or CUX1 knockdown.

Intuitively, one would assume that a transcription factor is more likely to regulate the closest promoter. Yet, some enhancers will exhibit an effect on a promoter situated on one side, but no effect on the promoter that is on the other side on the map. This sort of selectivity between an enhancer and a promoter has been explained by the presence of boundary or insulator elements or by specific interactions between proteins bound at the enhancer and the regulated promoter. Previous studies on CUX1 have all focused on genes that contain a CUX1 binding site within the immediate promoter. To begin to investigate the rules that govern the action of CUX1, we calculated the fraction of different types of CUX1 targets that were regulated in response to changes in CUX1 levels. Three types of genes were analyzed: 1, genes that are the closest to the CUX1 binding site; 2, genes that are further away and in the other direction from the CUX1 binding site; 3, genes that are located further away and are separated by another gene from the CUX1 binding site. For each category, we calculated the percentage of genes that exhibit a 1.25 or 1.5-fold change in expression following p110 CUX1 overexpression or CUX1 knockdown. Strikingly, essentially similar fractions of genes were regulated whether they were closest to the CUX1 binding site or were located further away in the other direction (Figure 5, compare 1 and 2). Moreover, the proportion of regulated genes was not significantly lower among genes that belong to the third category (Figure 5, type 3 genes). We conclude that CUX1 is capable of regulating genes at a distance. Moreover, CUX1 can regulate more than one gene on certain genomic loci.

## Discussion

Genome-wide location analysis on the ENCODE array revealed that ~47% of CUX1 binding sites are located in the 4-Kbp region upstream and downstream of a TSS, while more than 14% of CUX1 binding sites are situated at more than 40 Kbp from a TSS (Figure 2). Overall, 7.4% and 8.4% of putative targets on the ENCODE and promoter arrays respectively, exhibited a 1.5-fold change in expression following CUX1 knockdown or p110 CUX1 overexpression (Tables 15, 16, 17, 18 and 19). This proportion is within the 1-10% range of potential targets that have been reported to be regulated by other transcription factors [44-46].

**Table 18:** Number of genes on the promoter array that are regulated in response to CUX1 overexpression or CUX1 knockdown (1.25 fold)

**Effect of CUX1 on all genes and 4140 putative targets**	**CUX1 Overexpression or Knockdown**				**CUX1 Overexpression**		**CUX1 Knockdown**	
**Gene list**	**All genes**		**Target genes**		**Target genes**		**Target genes**	
Up- or Downregulated	4880	27.7%	1437	34.7%	568	13.7%	1083	26.1%
Upregulated	2290	13.0%	696	16.8%	261	6.3%	546	13.2%
Downregulated	2590	14.7%	744	17.9%	307	7.4%	537	13.0%

Number and percentage of all genes and CUX1 target genes on the promoter array that exhibit a 1.25 fold change in expression following p110 CUX1 overexpression or CUX1 knockdown. Genes were analyzed as in Table 15.

**Table 19:** Number of genes on the promoter array that are regulated in response to CUX1 overexpression or CUX1 knockdown (1.5 fold)

**Effect of CUX1 on all genes and 4140 putative targets**	**CUX1 Overexpression or Knockdown**				**CUX1 Overexpression**		**CUX1 Knockdown**	
**Gene list**	**All genes**		**Target genes**		**Target genes**		**Target genes**	
Up- or Downregulated	1231	7.0%	347	8.4%	85	2.1%	287	6.9%
Upregulated	591	3.4%	181	4.4%	28	0.7%	169	4.1%
Downregulated	640	3.6%	167	4.0%	57	1.4%	118	2.8%

Number and percentage of all genes and CUX1 target genes on the promoter array that exhibit a 1.5 fold change in expression following p110 CUX1 overexpression or CUX1 knockdown. "Upregulated by CUX1" are genes whose expression is increased following p110 CUX1 and/or decreased following CUX1 knockdown. Conversely, "Downregulated by CUX1" are genes whose expression is decreased following p110 CUX1 and/or increased following CUX1 knockdown. The total number of genes and target genes were 17586 and 4140, respectively.

Importantly, analysis of the percentage of regulated genes versus the distance of CUX1 binding sites to TSS showed that essentially the same proportion of genes are regulated whether CUX1 binds close or far away from the TSS (Figure 4A and B). In other words, the probability that a gene is regulated by CUX1 is not affected by the distance between the CUX1 binding site and the TSS. In addition, our results indicate that the position of genes relative to a CUX1 binding site do not determine whether these genes are regulated by CUX1. CUX1 regulated similar percentages of genes whether they were closest to the CUX1 binding site or were located further away in the other direction (Figure 5, compare 1 and 2). Moreover, CUX1 regulated a surprisingly high proportion (5.4%) of genes that were separated from their binding site by another gene (Figure 5). Altogether these results demonstrate that CUX1 can regulate genes at a distance and can regulate more than one gene on certain genomic loci.

The proportion of target genes that were found to be activated or repressed by CUX1, respectively 52% and 48% (Tables 18), is significantly different from what we reported in previous studies on target genes involved in cell cycle progression, cell motility, or the DNA damage response [21,22,57]. In each case, a vast majority of genes were found to be activated by p110 CUX1, whether we performed siRNA-mediated knockdown or overexpression of p110 CUX1. One factor that may explain this could be the functional classes of genes that were studied previously. The functional class of “cell cycle” genes includes mostly genes that stimulate cell cycle progression. Out of 25 cell cycle gene targets identified by ChIP-chip, 22 were activated and 2 were repressed by CUX1 (while only one was not affected) [21]. One of the two repressed genes, p21^WAF1/CKI1^, code for a CDK-inhibitor that blocks cell cycle progression, while the other, CCNH, is involved in transcription and DNA repair. All target genes that were activated play a positive role in cell cycle progression. Similarly, among 19 targets that play a role in DNA damage response, 18 were activated and one was repressed [57]. The repressed gene again was p21^WAF1/CKI1^. Overall, these results are consistent with the notion that CUX1 establishes a transcriptional program that promotes cell cycle progression and at the same time ensures the maintenance of genetic integrity.

We employed two experimental approaches to examine the transcriptional regulation of genes by CUX1. Expression profiling was performed following shRNA-mediated knockdown of CUX1 or p110 CUX1 overexpression. Among targets identified on the promoter array, 287 genes exhibited a 1.5-fold change in expression following CUX1 knockdown, while 85 genes were regulated in response to p110 CUX1 overexpression. Therefore, more genes were found to be regulated by CUX1 using the shRNA approach. This result can be interpreted to mean that CUX1 is required for optimal expression of many target genes, however, increasing CUX1 expression is not sufficient to modulate the expression of some target genes.

The CUX1 consensus binding site, ATCRAT (where R = C or A), was found to be present at 47.2% of the 5828 bound genomic sites (Table 8). We conclude that the presence of a CUX1 consensus binding site contributes to, but is not sufficient for, the recruitment of CUX1 to specific genomic locations. We envision that interactions with other transcription factors play an important role in recruiting CUX1 to specific locations. In agreement with this notion, functional analysis revealed distinct sets of cellular functions among gene targets that contain an ATCRAT consensus and those that do not (Tables 9 and 10). We note that functional classes involved in cell cycle were over-represented among target genes that do not contain a consensus CUX1 binding site (Table 10). In previous studies, CUX1 was shown to interact with E2F factors and cooperate with these factors in the regulation of several cell cycle genes [58,59]. It is likely that protein-protein interaction with E2F factors reduces the requirement for the presence of a high-affinity binding site for the recruitment of CUX1 on this class of genes.

CUX1 can be purified efficiently by immunoprecipitation or affinity chromatography. Following cross-linking, however, the yield of purification is drastically reduced such that we need 500 million cells to perform chromatin immunoprecipitation or affinity purification (ChIP or ChAP) for CUX1. This caveat has limited our ability to perform ChIP-sequencing and therefore our study relied on microarray hybridizations. While sequence coverage is admittedly smaller on microarrays, data collected from both ENCODE and promoter arrays have enabled us to define the importance of the CUX1 consensus binding site in the recruitment of CUX1 to genomic locations and determine whether CUX1 can regulate genes at a distance.

## Conclusions

Our results demonstrate that p110 CUX1 can mediate transcriptional repression or activation of specific genes when bound at variable distances from the transcription start site. Although the CUX1 consensus binding motif, ATCRAT, plays a role in the recruitment of CUX1 to specific genomic sites, protein-protein interactions must contribute to its transcriptional activity.

## Methods

### Cell culture

Hs578T is a human mammary carcinoma cell line [60]. Previous studies have documented changes in gene expression in response both to CUX1 knockdown and overexpression [21,33]. Hs578T cells were maintained in Dulbecco’s modified minimum essential medium (DMEM)(Wisent) supplemented with penicillin-streptomycin, and 5% fetal bovine serum (FBS) (Gibco).

### Retroviral infection and stable cell lines

Retroviruses were produced by transfecting 293VSV cells with the pREV/TRE vector either empty or encoding p110 CUX1-Tag^2^ (CUX1 a.a. 612–1336 with protein A and CBP tags inserted at the C-terminus) (Clontech). Preparation of the retroviruses and stable cell lines was done as previously described [37].

### Chromatin Affinity Purification (ChAP)

The method of chromatin affinity purification (ChAP) has previously been validated [47], and described in detail [48]. To ensure that the recombinant p110-Tag^2^ protein would be expressed at moderate level, we employed the pRevTRE retroviral vector (Clontech), which contains the minimal CMV promoter with a tetracycline responsive element. Importantly, no tetracycline was added to the medium. Moreover, the Hs578T breast tumor cells do not express a tetracycline-responsive transactivator. Basal expression from the pRevTRE vector was previously shown to be very low [61-63]. ChAP was performed on 5 x10^8^ Hs578T. The cell nuclei were purified as described in [64], then lysed in RIPA-M buffer (10 mM Tris–HCl pH8, 1 mM EDTA, 0.5 mM EGTA, 150 mM NaCl, 1% Triton X-100, 0.5% DOC, 0.1% SDS, 1 mM PMSF, protease inhibitors) and sonicated on ice to obtain 250- to 800-bp-long DNA fragments. Stably expressed recombinant p110-Tag^2^ protein was purified by the Taptag purification method with some modifications [65]. The IgG matrix bound p110-Tag^2^/DNA were washed in wash buffer I (20 mM Tris–HCl pH8, 2 mM EDTA, 2 mM EGTA, 150 mM NaCl, 1% NP-40, 0.5% DOC, 0.2% SDS), wash buffer II (20 mM Tris–HCl pH9, 2 mM EDTA, 2 mM EGTA, 500 mM NaCl, 1% NP-40, 0.5% DOC, 0.1% SDS), wash buffer III (50 mM Tris–HCl pH7.5, 2 mM EDTA, 1 mM EGTA, 0.5 M LiCl, 1% NP-40, 0.7% DOC,) and then TEV buffer (10 mM Tris–HCl (pH8.0), 100mMNaCl, 0.1% TX-100, 0.5 mM EDTA, 10% glycerol, 1 mM DTT). After TEV protease digestion, the released protein/DNA complexes were purified by affinity chromatography on calmodulin beads in the presence of calcium and then eluted with EGTA. After de-crosslinking, samples were treated with RNase A and Proteinase K. Un-enriched input chromatin was put aside as a control.

### Preparation of ChAP purified DNA for hybridization

ChAP purified chromatin was amplified by the method of Ligation-Mediated PCR as detailed previously [66]. Briefly, ChAPed DNAs and input DNA were blunted, ligated to a unidirectional linker and amplified by PCR for 24 cycles to generate a sufficient amount of DNA. Amplified DNA samples were Cy5 labeled and amplified input controls were Cy3 labeled using Nimblegen's Dual-Color DNA Labeling Kit according to the manufacturer's instructions.

### DNA microarray hybridization

Labeled samples were hybridized to either NimbleGen's HG17 ENCODE or their HG18 Human Promoter Array Set high density oligonucleotide tiling array (385 k probe format) and then washed according to the manufacturer's instructions. Arrays were scanned on an Agilent 5 μm scanner model G2505B using customized scan area settings (X: 28, Y:6, Width: 20, Height: 14, values in mm).

### ChAP-microarray result analysis

For both array platforms (Encode and promoter array), grid alignment, raw signal extraction, peak identification and peak mapping were carried out using the Nimblescan v8.0 software according to the company's instructions. Identified peaks were considered significant with a false discovery rate (FDR) below 0.05, which is considered highly confident. Further analysis of identified binding sites was carried our using either the R platform for statistical computing (http://www.R-project.org) or scripts written in PERL (Practical Extraction and Report Language, http://www.perl.org). All peaks identified in ChAP-Chip experiments on the Encode Array and the Human Promoter Array Set are provided (Additional files 3 and 4, respectively).

### ChAP-microarray result validation

Independent ChIP experiments using antibodies specific for endogenous CUX1 were carried out in Hs578t, as previously described [21]. Real-time PCR was used to measure the level of enrichment of genomic target regions in ChIP DNA vs. the un-enriched input DNA. We selected 25 genes from both the targets identified on the ENCODE array and on the Promoter array set and designed primers specific for the corresponding regions where CUX1 was putatively identified as binding.

### ENCODE binding sites for c-MYC and E2F1

We used ChIP-chip binding sites for E2F1 and c-Myc downloaded from the website of Dr. Peggy Farnham laboratory at <http://genomics.ucdavis.edu/farnham/suppdata.html>. This dataset contains the binding sites predicted for E2F1, c-MYC and POLR2A (RNA polymerase II) in the ENCODE regions classified by 4 criteria: L1 (P < 0.0001 and 98^th^ percentile), L2 (P < 0.0001 and 95^th^ percentile), L3 (P < 0.05 and 98^th^ percentile) and L4 (P < 0.05 and 95^th^ percentile) [14]. Based on the validation of 29 binding sites, Bieda et al. conclude that L1 binding sites are highly reliable, L2 and L3 binding sites are also reliable however based on sparser testing and L4 binding sites are usually artifacts. Binding sites identified with the L1 criteria were used for our analyses. The chromosomal intervals for binding sites predicted for E2F1 and POLR2A belonged to genomic coordinates using hg16, whereas c-MYC binding sites were in hg17. Therefore, the lift-over program found on the online GALAXY platform [67-69] was used and random results were verified using UCSC genome browser to convert hg16 coordinates to those of hg17. There were 1 and 2 binding sites for E2F1 at L1 and L3 respectively (hg16), which could not be mapped to hg17.

### DHS and ChromHMM data analysis

Data tracks were downloaded from the UCSC's Encode data portal (http://genome.ucsc.edu/ENCODE/). Genomic locations were compared to those of the CUX1 binding sites using scripts written in R. UCSC Accession numbers of the tracks used are: wgEncodeEH000503 (GEO accessions GSM736552 and GSM736634) for the DHS data and wgEncodeEH000786 for the ChromHMM data.

### Consensus sequence analysis

Genomic sequences corresponding to regions of interest (binding sites or other) were obtained using the online GALAXY platform. Scripts written in R were used to identify the ATCRAT consensus motif within regions of interest.

### De novo binding motif identification

De Novo motif discovery was performed using the DREME (Discriminative DNA Motif Discovery) motif discovery tools form the MEME suite of tools. Comparison with known DNA binding motifs was performed using the TOMTOM algorithm using the JASPAR CORE database as a reference for comparison. (meme.nbcr.net/) [70-72].

### Functional overrepresentation analysis

Identification of overrepresented gene functions was carried out using the online annotation tool DAVID. Genes that were bound by CUX1 (Targets) were compared with all genes present on the microarray (Background). Overrepresentation of a function depends on the increase in the proportion of genes involved in a given function between CUX1 targets and the background. The P-value is determined using an improved Fisher’s exact test from the DAVID software [73,74].

### p110 CUX1 overexpression and CUX1 shRNA

For overexpression, Hs578t cells were infected with a lentiviral vector expressing p110 CUX1. Duplicate infections were carried out in parallel and cells were harvested after 24 hours. For CUX1 knockdown, a stable Hs578t cell line containing a doxycycline inducible shRNA was established by retroviral infection. Doxycycline was applied to the cells for 6 days before harvest with control cells left untreated. Knockdown experiments were carried out in biological duplicates in parallel.

### Expression profiling sample preparation and hybridization

Total RNA was isolated from cells using the Arcturus Picopure RNA isolation kit. 2 μg of RNA was then amplified using the Arcturus RiboAmp PLUS RNA amplification kit according to the manufacturer's instructions for a single round of amplification. Amplified mRNA (aRNA) was labeled using the Arcturus Turbo Labelling Cy5 and Cy3 kits using the manufacturer's instructions with a modification: The labeling reaction was carried out using 5 μg of aRNA in a 20 μl volume instead of 50 μl to increase the dye incorporation rate. Labelled aRNA was hybridized to Agilent's Whole Human Genome Microarry (G4112F) according to the manufacturer's instructions, washed and scanned on a 5 μm Agilent scanner. Hybridizations of the biological duplicates of each experiment were carried out in technical duplicates using dye swaps (Cy3 and Cy5), for a total of 4 replicates for each of the overexpression and downregulation experiment.

### Expression profiling data analysis

Raw signal and background intensities were extracted from the scanned images of expression arrays using the Feature Extraction software from Agilent. Raw data was processed and normalized using the R platform and the LIMMA package [75]. Processed expression profiling results are provided (Additional file 5).

### Expression profiling result validation

Independent p110 CUX1 overexpression and CUX1 shRNA knockdown experiments were carried out in Hs578t cells using retroviral vectors. 10 genes were selected from each experiment and real-time PCR was used to confirm the changes in expression seen in expression profiling.

## Abbreviations

ChAP: Chromatin Affinity Purification; ChIP: Chromatin Immuno Precipitation; DHS: DNAse hypersensitivity site; ENCODE: ENCyclopedia of DNA Elements; FDR: False discovery rate; TAP: Tandem affinity purification; TSS: Transcription start site.

## Competing interests

The authors declare that they have no competing interests.

## Authors’ contributions

AN, CV and AA conceived the study. CV, AA, RH, PLC, LL, and GB performed the experiments. CV and AA performed data analysis. AN and CV wrote the manuscript. All authors read and approved the final manuscript.

## Supplementary Material

Additional file 1: Figure S1 Distribution of 3 random sets of binding sites relative to transcription start sites. Click here for file

Additional file 2: Figure S2 Distribution of binding sites relative to transcription start sites for 6 transcription factors. Click here for file

Additional file 3 CUX1 Binding site peaks from ENCODE array ChAP. Click here for file

Additional file 4 CUX1 Binding site peaks from Promoter array ChAP. Click here for file

Additional file 5 Expression profiling results following overexpression and knockdown of CUX1. Click here for file
