# Supplementary material for: Long-range transcriptional regulation by the p110 CUX1 homeodomain protein on the ENCODE array
Source: BMC Genomics. 2013 Apr 16;14:258. doi: 10.1186/1471-2164-14-258 (PMC3770232; doi:10.1186/1471-2164-14-258)
Supplement: Additional file 1: Figure S1 — Distribution of 3 random sets of binding sites relative to transcription start sites. [file 1471-2164-14-258-S1.pdf]

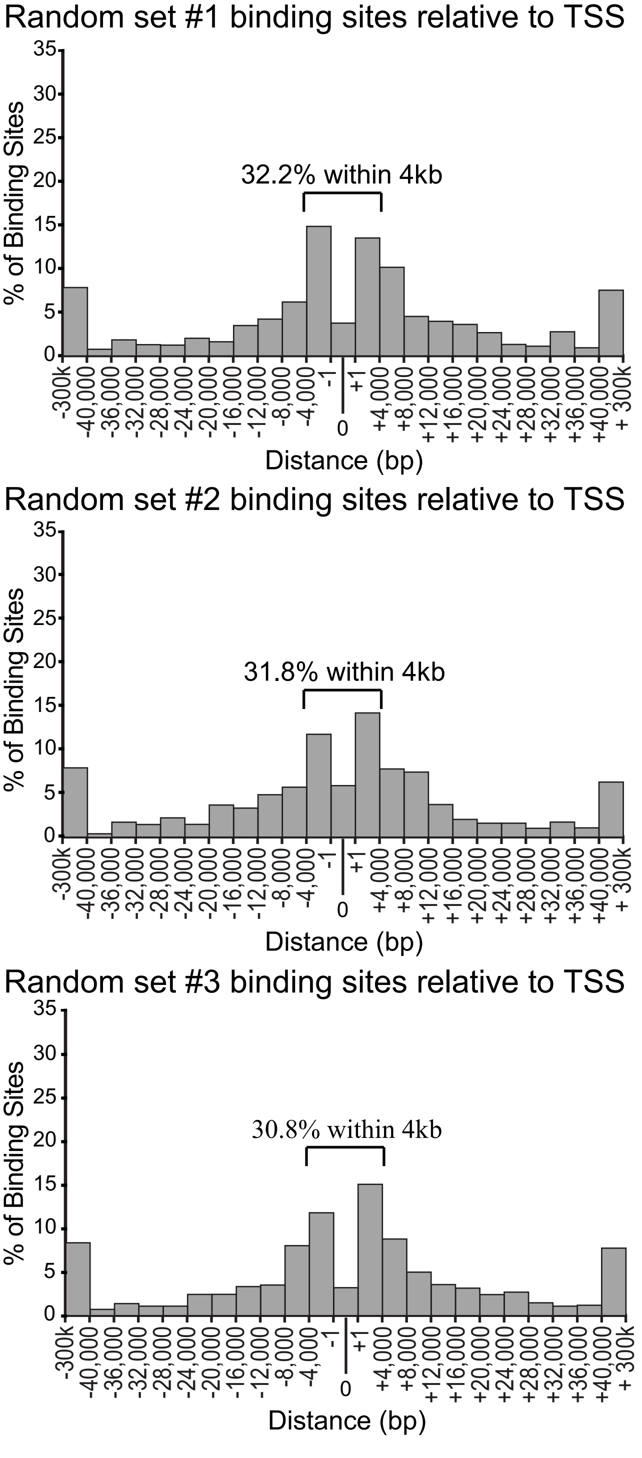

Additional Figure 1 – Distribution of 3 random sets of binding sites Relative to Transcription Start Sites
Percentage of Randomly generated binding sites located at various distances from the closest transcription start site. The "0" column indicates genes where the binding site overlaps the start site. 3 sets of 513 randomly located sites having the same distribution of sizes as CUX1 binding sites were generated.
