# Supplementary material for: Long-range transcriptional regulation by the p110 CUX1 homeodomain protein on the ENCODE array
Source: BMC Genomics. 2013 Apr 16;14:258. doi: 10.1186/1471-2164-14-258 (PMC3770232; doi:10.1186/1471-2164-14-258)
Supplement: Additional file 2: Figure S2 — Distribution of binding sites relative to transcription start sites for 6 transcription factors. [file 1471-2164-14-258-S2.pdf]

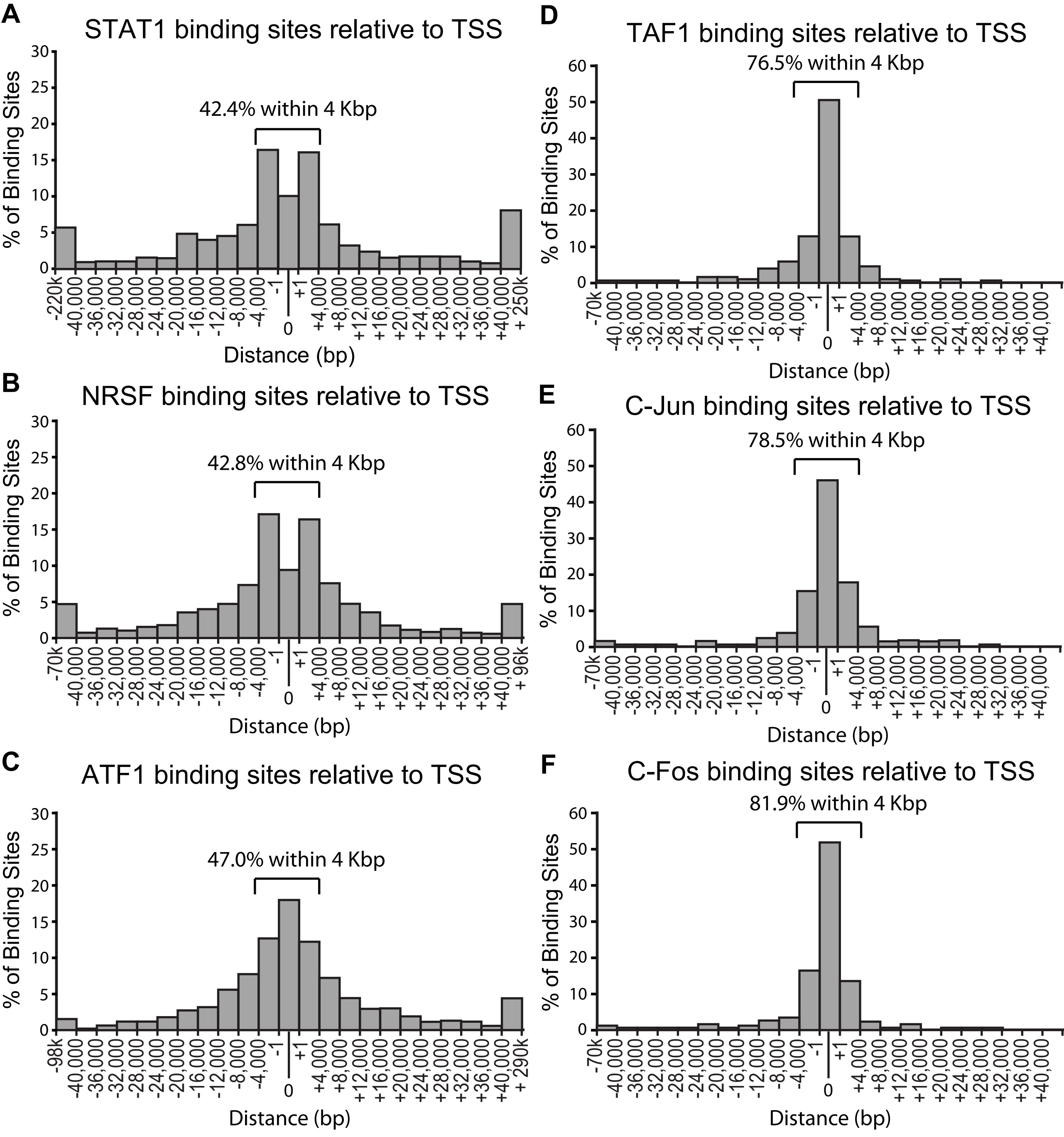

Additional Figure 2 – Distribution of binding sites Relative to Transcription Start Sites
Percentage of binding sites located at various distances from the closest transcription start site for 6 different transcription factors. The "0" column indicates genes where the binding site overlaps the start site.
